# Supplementary material for: A functional screen for bacterial ubiquitin regulation identifies an unusual Pseudomonas aeruginosa E3 ligase
Source: EMBO J. 2026 Sep 28;45(21):7489–514. doi: 10.1038/s44318-026-00917-7 (PMC13728172; doi:10.1038/s44318-026-00917-7)
Supplement: Supplementary file 1 [file 44318_2026_917_MOESM1_ESM.pdf]

THE EMBO JOURNAL

A functional screen for bacterial ubiquitin regulation identifies an unusual Pseudomonas aeruginosa E3 ligase

Cameron Roberts, Supender Kaur, Shun-Je Bhark, Aaron Ogden, Michael Divine, Gus Warren, Donghoon Kang, Natalia Kirienko, Paul Geurink, Monique Mulder, Ernesto Nakayasu, Jason McDermott, Joshua Adkins, Alejandro Aballay, and Jonathan Pruneda

Corresponding author(s): Jonathan Pruneda (pruneda@ohsu.edu)

Review Timeline:

Submission Date:	19th Sep 24
Editorial Decision:	7th Oct 24
Revision Received:	2nd Aug 26
Editorial Decision:	25th Aug 26
Revision Received:	10th Sep 26
Accepted:	11th Sep 26

Editor: Hartmut Vodermaier

Transaction Report:

(Note: With the exception of the correction of typographical or spelling errors that could be a source of ambiguity, letters and reports are not edited. Depending on transfer agreements, referee reports obtained elsewhere may or may not be included in this compilation. Referee reports are anonymous unless the Referee chooses to sign their reports.)

Dr. Jonathan N. Pruneda
Oregon Health & Science University
Molecular Microbiology & Immunology
3181 SW Sam Jackson Park Rd
Mail Code L220
Portland, OR 97239

7th Oct 2024

Re: EMBOJ-2024-119076
A functional screen for ubiquitin regulation identifies a Pseudomonas aeruginosa E3 ligase

Dear Jonathan,

Thank you again for submitting your study on identification of new bacterial ubiquitin regulators to The EMBO Journal. It has now been reviewed by three expert referees, whose reports are copied below. Since they all appreciate the quality and potential interest of the work, we shall be happy to consider a revised version further for publication. As you will see, the referees do bring up a number of well-taken queries and excellent suggestions, regarding the analyses and their presentation, which would be good to briefly go over early on, to define the directions of the revision work. Therefore, please do send me a tentative point-by-point response discussing the various general and specific comments of the reviewers, once you had a chance to go through them with your coworkers. As always, I would be happy to offer an extended revision period if this should be helpful in order to decisively address key referee concerns.

Detailed information on preparing, formatting and uploading a revised manuscript can be found below and in our Guide to Authors. Thank you again for the opportunity to consider this work for The EMBO Journal, and I look forward to hearing from you in due time.

With kind regards,

Hartmut

Hartmut Vodermaier, PhD
Senior Editor, The EMBO Journal
h.vodermaier@embojournal.org

*** PLEASE NOTE: All revised manuscript are subject to initial checks for completeness and adherence to our formatting guidelines. Revisions may be returned to the authors and delayed in their editorial re-evaluation if they fail to comply to the following requirements (see also our Guide to Authors for further information):

1) Every manuscript requires a Data Availability section (even if only stating that no deposited datasets are included). Primary datasets or computer code produced in the current study have to be deposited in appropriate public repositories prior to resubmission, and reviewer access details provided in case that public access is not yet allowed. Further information: embopress.org/page/journal/14602075/authorguide#dataavailability

2) Each figure legend must specify
- size of the scale bars that are mandatory for all micrograph panels
- the statistical test used to generate error bars and P-values
- the type error bars (e.g., S.E.M., S.D.)
- the number (n) and nature (biological or technical replicate) of independent experiments underlying each data point
- Figures may not include error bars for experiments with n<3; scatter plots showing individual data points should be used instead.

3) Revised manuscript text (including main tables, and figure legends for main and EV figures) has to be submitted as editable text file (e.g., .docx format). We encourage highlighting of changes (e.g., via text color) for the referees' reference.

4) Each main and each Expanded View (EV) figure should be uploaded as individual production-quality files (preferably in .eps, .tif, .jpg formats). For suggestions on figure preparation/layout, please refer to our Figure Preparation Guidelines: http://bit.ly/EMBOPressFigurePreparationGuideline

5) Point-by-point response letters should include the original referee comments in full together with your detailed responses to them (and to specific editor requests if applicable), and also be uploaded as editable (e.g., .docx) text files.

6) Please complete our Author Checklist, and make sure that information entered into the checklist is also reflected in the manuscript; the checklist will be available to readers as part of the Review Process File. A download link is found at the top of our Guide to Authors: embopress.org/page/journal/14602075/authorguide

7) All authors listed as (co-)corresponding need to deposit, in their respective author profiles in our submission system, a unique ORCiD identifier linked to their name. Please see our Guide to Authors for detailed instructions.

8) Please note that supplementary information at EMBO Press has been superseded by the 'Expanded View' for inclusion of additional figures, tables, movies or datasets; with up to five EV Figures being typeset and directly accessible in the HTML version of the article. For details and guidance, please refer to:
embopress.org/page/journal/14602075/authorguide#expandedview

9) To facilitate reproducibilty and cross-laboratory adoption of methodologies, please structure the Materials & Methods section as outlined in our guide to authors, including a completed Reagents and Tools Table that can be downloaded from our author guidelines as well (https://www.embopress.org/page/journal/14602075/authorguide#structuredmethods).

10) Digital image enhancement is acceptable practice, as long as it accurately represents the original data and conforms to community standards. If a figure has been subjected to significant electronic manipulation, this must be clearly noted in the figure legend and/or the 'Materials and Methods' section. The editors reserve the right to request original versions of figures and the original images that were used to assemble the figure. Finally, we generally encourage uploading of numerical as well as gel/blot image source data; for details see: embopress.org/page/journal/14602075/authorguide#sourcedata

At EMBO Press, we ask authors to provide source data for the main manuscript figures. Our source data coordinator will contact you to discuss which figure panels we would need source data for and will also provide you with helpful tips on how to upload and organize the files.

Further information is available in our Guide For Authors:
https://www.embopress.org/page/journal/14602075/authorguide

In the interest of ensuring the conceptual advance provided by the work, we recommend submitting a revision within 3 months (5th Jan 2025). Please discuss the revision progress ahead of this time with the editor if you require more time to complete the revisions. Use the link below to submit your revision:

Link Not Available

Referee #1:

The manuscript by Roberts et al developed a workflow to screen for bacterial effectors that directly target ubiquitin, and subsequently identified PUL-1, a novel E3 ubiquitin ligase secreted by Pseudomonas aeruginosa, which exhibits robust activity in synthesizing nonspecific polyUb chains. Additionally, PUL-1 displays a fold highly similar to that of acyl-CoA dehydrogenases, distinct from known E3 ubiquitin ligases, with Cys4 serving as the key catalytic residue. Interestingly, homologs of PUL-1 are found in other bacteria, all of which share a similar fold and conserved catalytic residue, suggesting that PUL-1 represents a novel class of E3 ubiquitin ligases widely distributed among various pathogenic bacteria. Compared to the wild-type infection group, worms infected with the pul-1Tn strain exhibit a higher bacterial burden and severe intestinal bloating, accompanied by a significant reduction in lifespan, indicating a certain physiological function.

This manuscript is of potential interest, and many points are well-supported. The identification of a previously unrecognized family of E3 ubiquitin ligases that PUL-1 represents is particularly original.

Here are some suggestions for improvement:

(1) The secretion assay in this study relies on EGTA stimulation. This may not be a physiologically relevant event. Experiments showing that Ubiquitination of proteins can occur during Pseudomonas aeruginosa infection, at levels secreted by bacteria, are needed to confirm this activity.
(2) Does the dehydrogenase activity depend on the autoubiquitination of PUL-1?
(3) The authors identified Cys4 as the key catalytic residue of PUL-1 through SASA analysis, but further biochemical

experiments are needed to confirm that other cysteines are not involved.
(4) Fig. S5C can be improved by displaying multiple sequence alignment result and highlighting the conserved Cys4 residue.
(5) It remains to be determined whether homologs of PUL-1 in other bacteria possess E3 ubiquitin ligase activity and whether the conserved Cys or Ser functions as the catalytic residue. Additionally, Fig. S5D shows that homologs from Pseudomonas fluorescens (WP_039768646.1) and Pseudomonas putida (WP_019437599.1) lack conserved Cys or Ser residues, instead having conserved Asn. Therefore, it is unclear whether they possess E3 ubiquitin ligase activity.
(6) Do acyl-CoA dehydrogenases in eukaryotic cells have conserved Cys or Ser residues, and if so, do they possess E3 ubiquitin ligase activity?
(7) In Fig. 6C, why is there still a significant difference in bacterial burden between the complemented strain pul-1Tn::pul-1 and the wild type? What could be the possible reasons for this?

Referee #2:

The MS by Roberts et al. reported a non-biased strategy to identify ubiquitin regulators from secreted bacterial effectors. Applying this approach, the authors reported the identification of a cryptic E3 ligase secreted by Pseudomonas aeruginosa, which they named PUL-1. PUL-1 adopts an acyl-CoA dehydrogenase fold but lacks the dehydrogenase activity against an octanoyl-CoA substrate. The authors further mapped the catalytic site to residue C4. Surprisingly the authors showed that PUL-1 exhibited more of an antivirulence role using C-elegans as the infection model. Overall, this is a well-written story. The unbiased approach to identifying ubiquitin modulators is a useful tool in some cases, particularly for those unusual ubiquitin modulators that escaped any computational prediction. However, this approach has a limited broad impact in that there are likely not many unknown ubiquitin regulators to be discovered.
Major and minor comments:
1. Most of the biochemical assays are solid and convincing. However, in Fig. 4F. the discharging of PUL-1~Ub by lys is not obvious. It probably needs several repeats and quantification.
2. Fig 6C. pul-1Tn::pul-1 is supposed to have a similar effect as the WT, but the statistics showed a significant difference compared to the WT. What does this mean?
3. The characterization of PUL-1 as a ubiquitin E3 ligase is not sufficient. Particularly, how this E3 ligase recognizes E2s is not explained or sufficiently discussed.
4. It is quite surprising that C4 is the catalytic residue. In E3 ligases, the catalytic cysteine needs a certain chemical environment to be activated for Ub transthiolation. However, the amino terminus of a protein is supposed to be highly flexible, which makes it hard to keep C4 in an activated environment. How is the N-terminus of the protein stabilized in the predicted structure? Would perturbations to some key interactions at the N-terminus of the protein inactivate the enzyme?

Referee #3:

In this manuscript, Roberts, Kaur, Ogden et al. present a novel unbiased functional screening method to identify bacterial ubiquitin regulators. Using this approach, they discover a new E3 ubiquitin ligase in Pseudomonas aeruginosa, which they name Pseudomonas Ub ligase 1 (PUL-1). Through comprehensive biochemical and structural analyses, the authors demonstrate that PUL-1 represents a new family of E3 ligases with a fold similar to acyl-CoA dehydrogenases. They show that PUL-1's E3 ligase activity depends on a catalytic cysteine residue (Cys4) and that it can work with multiple E2 enzymes to form various types of polyubiquitin chains. Importantly, they demonstrate that PUL-1's ligase activity modulates P. aeruginosa virulence in a Caenorhabditis elegans infection model.
As a reviewer, I find the functional screening approach innovative and the biochemical characterization of PUL-1 thorough and convincing. The study provides valuable insights into a novel E3 ligase and advances our understanding of how bacterial pathogens can manipulate host ubiquitin systems. Overall, this is an intriguing story that introduces a new method for identifying bacterial ubiquitin regulators and expands our knowledge of bacterial E3 ligases.
However, I would ask the authors to address the following points in a revised manuscript to strengthen their findings:

[Major comments]
In general, this reviewer does not find it necessary to request any additional experiments from the authors. However, I recommend the authors re-format their manuscript as suggested below.

1. The manuscript's focus needs clarification. Currently, it appears to have two aims: developing a novel screening method and characterizing a new ubiquitin regulator in P. aeruginosa. I recommend that the authors choose one of the following approaches:

a) Focus more on the method development, using PUL-1 as a case study. This would involve expanding on the method's applicability to other bacterial species and potentially identifying additional examples of novel ubiquitin regulators.
b) Focus more on the P. aeruginosa story. This could be framed as: "No ubiquitin regulators had been found in P. aeruginosa. To discover them, we developed a novel unbiased method, which identified the PUL-1 E3 ligase."
If the authors choose to focus on the method (option a), they should expand their examples beyond P. aeruginosa to demonstrate the method's broad applicability.

If focusing on the P. aeruginosa story (option b), the authors should provide more context on why identifying ubiquitin regulators in this pathogen is particularly significant.
The current abstract emphasizes developing a new method, while 90% of the manuscript relates to PUL-1's activity, structure, putative mechanism, and virulence. Therefore, it seems there are not enough validation cases except for PUL-1.

2. Authors need to clarify the flow of their screening method. The authors showed that this novel screening method can detect both DUBs and E3 ligases. However, there is no description of why authors focused on the E3 ligase activity of the P. aeruginosa secretion fraction. Did the authors test the secreted fraction on the Ub-KG(Tamra) substrate? If yes, how would authors proceed to characterize this novel DUB(s)?

I am asking this specific question because the authors very carefully examined the PUL-1 ligase activities. Regarding point 1, authors can incorporate their workflow in the later part of the screening method. For example, discovery of a novel E3-ligase would follow: 1) Tamra-Ub assay, 2) Strain screening, 3) Target-ID by MS, 4) E2 screening, 5) UbiCRest assay and so on. I understand that these methods are already well-established, but by including these assays in Fig. 1A, authors can emphasize that their method is systematically well-designed and that following this led them to identify and characterize PUL-1. Regarding this, I am still wondering what happened when they mixed the secreted fraction with Ub-KG? And what would be the systematic approaches for a novel DUB? Authors do not need to describe all the current methods but can add a few standard methods that could be used once they find DUBs.

[Minor comments]

1. Have you tried using AF3 and analyze/judge its structure? I know that AF3 is still not perfect to use to guess novel complex structures, but the authors can test.

2.The PUL-1 structure seems highly similar to short-chain specific acyl-CoA dehydrogenase while others did not show the Ub-ligase activity. Since authors identified Cys4 as catalytic residue of PUL-1, I am wondering if this Cys is conserved in these SCAD enzymes that authors found.

3. Fig1G, H, Fig 2B and other graphs, Does 'no effectors' mean Lysate fraction? What happens when authors use lysate fraction? I know that lysate fraction is not the aim of this screening, but authors showed comparison of S & L in Ub-KG assay in Fig 1D, F. Specify the meaning of no effectors.

4. Though UbiCRest is an established method. Authors who are new to this field may wonder what is the meaning of the asterisk (*) in OTUB1 and AMSHin Figure 4E.

5. Fig S2C, no label for the red and black lines.

Re: EMBOJ-2024-119076
A functional screen for ubiquitin regulation identifies a Pseudomonas aeruginosa E3 ligase

Dear Jonathan,

Thank you again for submitting your study on identification of new bacterial ubiquitin regulators to The EMBO Journal. It has now been reviewed by three expert referees, whose reports are copied below. Since they all appreciate the quality and potential interest of the work, we shall be happy to consider a revised version further for publication. As you will see, the referees do bring up a number of well-taken queries and excellent suggestions, regarding the analyses and their presentation, which would be good to briefly go over early on, to define the directions of the revision work. Therefore, please do send me a tentative point-by-point response discussing the various general and specific comments of the reviewers, once you had a chance to go through them with your coworkers. As always, I would be happy to offer an extended revision period if this should be helpful in order to decisively address key referee concerns.

Detailed information on preparing, formatting and uploading a revised manuscript can be found below and in our Guide to Authors. Thank you again for the opportunity to consider this work for The EMBO Journal, and I look forward to hearing from you in due time.

With kind regards,

Hartmut

Hartmut Vodermaier, PhD
Senior Editor, The EMBO Journal
h.vodermaier@embojournal.org

We would like to thank all of the reviewers for their constructive feedback and overall positive outlooks on our work. We were pleased to see that the novelty of our approach as well as the identification of a novel E3 ligase in *Pseudomonas aeruginosa* were appreciated.

Referee #1:

The manuscript by Roberts et al developed a workflow to screen for bacterial effectors that directly target ubiquitin, and subsequently identified PUL-1, a novel E3 ubiquitin ligase secreted by Pseudomonas aeruginosa, which exhibits robust activity in synthesizing nonspecific polyUb chains. Additionally, PUL-1 displays a fold highly similar to that of acyl-CoA dehydrogenases, distinct from known E3 ubiquitin ligases, with Cys4 serving as the key catalytic residue. Interestingly, homologs of PUL-1 are found in other bacteria, all of which share a similar fold and conserved catalytic residue, suggesting that PUL-1 represents a novel class of E3 ubiquitin ligases widely distributed among various pathogenic bacteria. Compared to the wild-type

infection group, worms infected with the pul-1Tn strain exhibit a higher bacterial burden and severe intestinal bloating, accompanied by a significant reduction in lifespan, indicating a certain physiological function.

This manuscript is of potential interest, and many points are well-supported. The identification of a previously unrecognized family of E3 ubiquitin ligases that PUL-1 represents is particularly original.

Here are some suggestions for improvement:

(1) The secretion assay in this study relies on EGTA stimulation. This may not be a physiologically relevant event. Experiments showing that Ubiquitination of proteins can occur during Pseudomonas aeruginosa infection, at levels secreted by bacteria, are needed to confirm this activity.

Low calcium concentration is a known environmental cue sensed by *P. aeruginosa* for activation of the Type III Secretion System (McCaw et al., 2002). Thus, addition of EGTA activates a physiologically relevant pathway for effector secretion and, as such, is a widely utilized method in the *P. aeruginosa* field. We have improved the description of this approach in the text to reflect this.

Confirmation of PUL-1-dependent protein ubiquitination during infection first requires identification of a substrate, which is a considerable amount of work that is outside the scope of this study. We have, however, demonstrated in the *C. elegans* system that the observed phenotype depends upon the PUL-1 catalytic cysteine, which is consistent with the E3 ligase activity of natively secreted PUL-1 playing a functional role within the eukaryotic host.

(2) Does the dehydrogenase activity depend on the autoubiquitination of PUL-1?

While this is an interesting question, we feel that it is outside of the scope of our study. Testing this idea properly would require the development of an approach to purify homogeneously auto-ubiquitinated PUL-1, which is not trivial. Given that related enzymes, such as FadE13, are fully capable of exhibiting dehydrogenase activity in the absence of ubiquitin, it would be surprising if PUL-1 had this additional layer of regulation.

(3) The authors identified Cys4 as the key catalytic residue of PUL-1 through SASA analysis, but further biochemical experiments are needed to confirm that other cysteines are not involved.

Considering that the remaining Cys residues are buried in the predicted PUL-1 structure, we are concerned that their mutation would lead to structural changes that could have indirect effects on PUL-1 ligase activity. Because we were able to both A) Eliminate PUL-1 ligase activity with a C4A mutation, and B) Capture a DTT-resistant but base-sensitive PUL-1~Ub intermediate with a C4S mutation, we feel that conclusive evidence is provided for identification of the active site.

(4) Fig. S5C can be improved by displaying multiple sequence alignment result and highlighting the conserved Cys4 residue.

The PUL-1 genes presented in Fig. S5C are perfectly conserved outside of the indicated amino acid substitutions, and thus we felt that a full sequence alignment would be unnecessary. We have clarified this in the figure legend.

(5) It remains to be determined whether homologs of PUL-1 in other bacteria possess E3 ubiquitin ligase activity and whether the conserved Cys or Ser functions as the catalytic residue. Additionally, Fig. S5D shows that homologs from Pseudomonas fluorescens (WP_039768646.1) and Pseudomonas putida (WP_019437599.1) lack conserved Cys or Ser residues, instead having conserved Asn. Therefore, it is unclear whether they possess E3 ubiquitin ligase activity.

Expansion of the PUL-1 ligase family into other organisms will indeed be an exciting area of future work. Already, given the 99% sequence identity (i.e., only a single His to Gln substitution distant from the ligase active site), we can predict with high confidence that the PUL-1 orthologue in *Acinetobacter baumannii* will function as an E3 ligase. Both *P. aeruginosa* and *A. baumannii* are classified as ESKAPE pathogens; so categorized for their high level of health relevance and risk of antimicrobial resistance. This finding clearly highlights the importance of studying the *A. baumannii* PUL-1 orthologue in future work. The orthologues that we highlighted as lacking the conserved Cys/Ser residue were included as demonstration that ligase activity is not conserved across all related genes. To better illustrate the degree of conservation, we have included the percent identity with respect to PAO1 PUL-1.

(6) Do acyl-CoA dehydrogenases in eukaryotic cells have conserved Cys or Ser residues, and if so, do they possess E3 ubiquitin ligase activity?

Again, this is a very interesting extension of our work, but we feel that it moves outside the scope of our study focused on the identification of new bacterial effectors in *P. aeruginosa* that regulate host ubiquitination.

(7) In Fig. 6C, why is there still a significant difference in bacterial burden between the complemented strain pul-1Tn::pul-1and the wild type? What could be the possible reasons for this?

It is difficult to say why this complementation did not provide a perfect rescue. It could be a result of our approach to complement *pul-1* on a plasmid, as noted in the text. Although we used the native promoter region for *pul-1* in this construct, the differing context could have altered its level of expression to some extent.

Referee #2:

The MS by Roberts et al. reported a non-biased strategy to identify ubiquitin regulators from secreted bacterial effectors. Applying this approach, the authors reported the identification of a cryptic E3 ligase secreted by Pseudomonas aeruginosa, which they named PUL-1. PUL-1 adopts

an acyl-CoA dehydrogenase fold but lacks the dehydrogenase activity against an octanoyl-CoA substrate. The authors further mapped the catalytic site to residue C4. Surprisingly the authors showed that PUL-1 exhibited more of an antivirulence role using C-elegans as the infection model. Overall, this is a well-written story. The unbiased approach to identifying ubiquitin modulators is a useful tool in some cases, particularly for those unusual ubiquitin modulators that escaped any computational prediction. However, this approach has a limited broad impact in that there are likely not many unknown ubiquitin regulators to be discovered.

We thank the reviewer for their observations, but respectfully disagree with their prediction that most ubiquitin regulators have been discovered by now. Even from the limited focus on a handful bacterial pathogens studied thus far (i.e., *Salmonella* Typhimurium, *Shigella flexneri*, *Escherichia coli*, and *Legionella pneumophila*), it is clear that the vast majority of bacterial E3 ligases have arisen through convergent evolution. This opens huge opportunities for discoveries in underexplored bacteria (e.g., *Mycobacterium tuberculosis*, *Listeria monocytogenes*, *Bordetella pertussis*, and many others) that have major impacts on human health, but encode no predicted E3 ligases based on sequence similarity. We have revised the Discussion section to emphasize that this approach will benefit future study of pathogens that have not been explored through the lens of ubiquitin signaling.

Major and minor comments:

1. Most of the biochemical assays are solid and convincing. However, in Fig. 4F. the discharging of PUL-1~Ub by lys is not obvious. It probably needs several repeats and quantification.

Considering the redundancy between this assay and our mass spectrometry analysis of modified sites, we have opted to remove the amino acid discharge experiment.

2. Fig 6C. pul-1Tn::pul-1 is supposed to have a similar effect as the WT, but the statistics showed a significant difference compared to the WT. What does this mean?

As we explain above, it is difficult to say why this complementation did not provide a perfect rescue. It could be a result of our approach to complement *pul-1* on a plasmid, as described in the text. Although we used the native promoter region for *pul-1* in this construct, the differing context could have altered its level of expression to some extent.

3. The characterization of PUL-1 as a ubiquitin E3 ligase is not sufficient. Particularly, how this E3 ligase recognizes E2s is not explained or sufficiently discussed.

While a full mechanistic characterization of PUL-1 ligase function will require follow-up studies, we have performed some modeling and mutational analyses that indicate PUL-1 interacts with the common E3-binding surface of UBE2D3. While not with high confidence, which is to be expected considering the unprecedented and cross-species nature of the PUL1:UBE2D3 complex, AlphaFold3 does model an interaction that spatially orients the two catalytic cysteines, utilizing the typical Helix 1, Loop 4, and Loop 7 region of UBE2D3 known to interact with other E3 ligases (new Fig. S5E). Mutational analysis is consistent with this model, as indicated by decreased PUL-1 ligase activity observed with an A96D mutation located in Loop 7 of UBE2D3 (new Fig. S5F). Further, interaction with this surface of UBE2D3 is

consistent with other E3 ligases that display a similar E2 preference as observed with PUL-1 (Fig. 4D).

4. It is quite surprising that C4 is the catalytic residue. In E3 ligases, the catalytic cysteine needs a certain chemical environment to be activated for Ub transthiolation. However, the amino terminus of a protein is supposed to be highly flexible, which makes it hard to keep C4 in an activated environment. How is the N-terminus of the protein stabilized in the predicted structure? Would perturbations to some key interactions at the N-terminus of the protein inactivate the enzyme?

These are interesting questions. It is worth noting that our basis of understanding for E3 ligase mechanism is rooted in a select group of ligases (primarily eukaryotic), and PUL-1 may or may not follow the same set of "rules". Further, much of the work focusing on the active site environment in established E3 ligases is centered around their ability to form specific polyubiquitin chains, and less so in their ability to simply transfer ubiquitin. Given the lack of polyubiquitin specificity exhibited by PUL-1, this could be consistent with a very minimal active site environment. Still, we think that this question will be an important topic of future research, along with other aspects of PUL-1 catalytic mechanism as discussed above.

Referee #3:

In this manuscript, Roberts, Kaur, Ogden et al. present a novel unbiased functional screening method to identify bacterial ubiquitin regulators. Using this approach, they discover a new E3 ubiquitin ligase in Pseudomonas aeruginosa, which they name Pseudomonas Ub ligase 1 (PUL-1). Through comprehensive biochemical and structural analyses, the authors demonstrate that PUL-1 represents a new family of E3 ligases with a fold similar to acyl-CoA dehydrogenases. They show that PUL-1's E3 ligase activity depends on a catalytic cysteine residue (Cys4) and that it can work with multiple E2 enzymes to form various types of polyubiquitin chains. Importantly, they demonstrate that PUL-1's ligase activity modulates P. aeruginosa virulence in a Caenorhabditis elegans infection model.
As a reviewer, I find the functional screening approach innovative and the biochemical characterization of PUL-1 thorough and convincing. The study provides valuable insights into a novel E3 ligase and advances our understanding of how bacterial pathogens can manipulate host ubiquitin systems. Overall, this is an intriguing story that introduces a new method for identifying bacterial ubiquitin regulators and expands our knowledge of bacterial E3 ligases. However, I would ask the authors to address the following points in a revised manuscript to strengthen their findings:

[Major comments]
In general, this reviewer does not find it necessary to request any additional experiments from the authors. However, I recommend the authors re-format their manuscript as suggested below.

1. The manuscript's focus needs clarification. Currently, it appears to have two aims: developing a novel screening method and characterizing a new ubiquitin regulator in P. aeruginosa. I

recommend that the authors choose one of the following approaches:

a) Focus more on the method development, using PUL-1 as a case study. This would involve expanding on the method's applicability to other bacterial species and potentially identifying additional examples of novel ubiquitin regulators.
b) Focus more on the P. aeruginosa story. This could be framed as: "No ubiquitin regulators had been found in P. aeruginosa. To discover them, we developed a novel unbiased method, which identified the PUL-1 E3 ligase."
If the authors choose to focus on the method (option a), they should expand their examples beyond P. aeruginosa to demonstrate the method's broad applicability.
If focusing on the P. aeruginosa story (option b), the authors should provide more context on why identifying ubiquitin regulators in this pathogen is particularly significant.
The current abstract emphasizes developing a new method, while 90% of the manuscript relates to PUL-1's activity, structure, putative mechanism, and virulence. Therefore, it seems there are not enough validation cases except for PUL-1.

We would like to thank the reviewer for this advice on how to balance the two primary aspects of our work. In this instance, our study was focused on *P. aeruginosa* as a pathogen with no known (or predicted) ubiquitin regulators, despite having a large repertoire of other virulence factors that manipulate host cell biology. As such, we are inclined to agree with the reviewer's second suggestion, and have revised the abstract and introduction accordingly.

2. Authors need to clarify the flow of their screening method. The authors showed that this novel screening method can detect both DUBs and E3 ligases. However, there is no description of why authors focused on the E3 ligase activity of the P. aeruginosa secretion fraction. Did the authors test the secreted fraction on the Ub-KG(Tamra) substrate? If yes, how would authors proceed to characterize this novel DUB(s)?

I am asking this specific question because the authors very carefully examined the PUL-1 ligase activities. Regarding point 1, authors can incorporate their workflow in the later part of the screening method. For example, discovery of a novel E3-ligase would follow: 1) Tamra-Ub assay, 2) Strain screening, 3) Target-ID by MS, 4) E2 screening, 5) UbiCRest assay and so on. I understand that these methods are already well-established, but by including these assays in Fig. 1A, authors can emphasize that their method is systematically well-designed and that following this led them to identify and characterize PUL-1.
Regarding this, I am still wondering what happened when they mixed the secreted fraction with Ub-KG? And what would be the systematic approaches for a novel DUB? Authors do not need to describe all the current methods but can add a few standard methods that could be used once they find DUBs.

To address the first point raised by the reviewer, we have now included data showing that we could not detect any DUB activity in *P. aeruginosa* using our approach (new Fig. S2A). We like the reviewer's suggestion of expanding our ideas into a recommended workflow for identification of ubiquitin-regulating effectors. To incorporate this, we have expanded the description of our workflow in the Discussion section and generated a graphical abstract that illustrates the approach.

[Minor comments]

1. Have you tried using AF3 and analyze/judge its structure? I know that AF3 is still not perfect to use to guess novel complex structures, but the authors can test.

Yes, we obtain very similar structural models for PUL-1 whether we use standard homology modeling, RoseTTAFold, AlphaFold2, or AlphaFold3.

2.The PUL-1 structure seems highly similar to short-chain specific acyl-CoA dehydrogenase while others did not show the Ub-ligase activity. Since authors identified Cys4 as catalytic residue of PUL-1, I am wondering if this Cys is conserved in these SCAD enzymes that authors found.

No, the catalytic Cys of PUL-1 is not conserved in the structurally homologous enzymes presented from rat or *B. thailandensis*, nor *M. tuberculosis* FadE13, which lacks E3 ligase activity. We have now included this observation in the text.

3. Fig1G, H, Fig 2B and other graphs, Does 'no effectors' mean Lysate fraction? What happens when authors use lysate fraction? I know that lysate fraction is not the aim of this screening, but authors showed comparison of S & L in Ub-KG assay in Fig 1D, F. Specify the meaning of no effectors.

We included lysates into the experiments presented in Figures 1D and F for comparison to the effector pools generated from secretion-deficient bacterial strains, thereby illustrating that blockage of secretion led to retention of the ligase/DUB activity in the lysate fraction. Subsequent experiments (including Figures 1G, H, and 2B) focused exclusively on the secreted fraction, and thus ‘no effectors’ should actually indicate ‘no addition’. We have clarified this in the revised figures.

4. Though UbiCRest is an established method. Authors who are new to this field may wonder what is the meaning of the asterisk (*) in OTUB1 and AMSH in Figure 4E.

We have clarified this nomenclature in the figure legend and methods.

5. Fig S2C, no label for the red and black lines.

We have incorporated these labels into the revised figure.

Dr. Jonathan N Pruneda
Oregon Health & Science University
Molecular Microbiology & Immunology
3181 SW Sam Jackson Park Rd
Mail Code L220
Portland, OR 97239

25th Aug 2026

Re: EMBOJ-2024-119076R
A functional screen for ubiquitin regulation identifies a Pseudomonas aeruginosa E3 ligase

Dear Jonathan,

Thank you for submitting your revised manuscript on bacterial ubiquitin regulator screening to The EMBO Journal. All three original referees have now reviewed it once more, and I am happy to say were fully satisfied with the revisions. Following addressing of a number of editorial points, as listed below, we should therefore be ready to proceed with formal acceptance and publication of the study:

- Importantly, our routine pre-acceptance image checks indicated that certain worm micrographs are re-displayed in Figures 6D and EV5E, although this is not explicitly mentioned and rationalized in the respective figure legends - please carefully double-check and, if warranted, explain in both respective figure legends

- Please enter valid email addresses for all coauthors in the submission system, so that they could be informed about the submission and final decision. At resubmission, acknowledgement emails failed to be delivered to Supender Kaur and Donghoon Kang.

- Please adjust the order of the manuscript sections, and also make sure to use the correct section headers: Title page with complete author information, Abstract, Introduction, Results, Discussion, Methods, Data Availability, Acknowledgements, Disclosure and Competing Interests Statement, References, Main Figure Legends, Tables, Expanded Figure Legends.

- Please double-check to make sure to all relevant funding information in the manuscript is congruent with the info entered into our submission system. Currently missing in the submission system are: Oregon Health & Science University (JNP)

- As we have switched from a free-text author contribution statement towards a more formal statement based on Contributor Role Taxonomy (CRediT) terms, please remove the present Author Contribution section and instead specify each author's contribution(s) directly in the Author Information page of our submission system during upload of the final manuscript. See https://casrai.org/credit/ for more information.

- Please adjust the format of the reference list and of the in-text citations according to EMBO Journal format (alphabetical order, author name et al + year...), and make sure that each reference is complete with citation year, volume, and page/elocator numbers. Please include DOI information/URLs only in case of advance publications that do not yet have volume/page numbers.

- Referee 1 notes that some figure labels warrant double-checking (see comments below).

- Please upload the included Source Data according to the scheme 'one ZIP archive per main figure', and 'one combined ZIP archive for all EV figures'.

- Please provide suggestions for a short 'blurb' text prefacing and summing up the conceptual aspect of the study in two sentences (max. 250 characters), followed by 3-5 one-sentence 'bullet points' with brief factual statements of key results of the paper; they will form the basis of an editor-written 'Synopsis' accompanying the online version of the article. Please also provide a simpler synopsis image (for use as a "visual title"), ideally in JPG format and in the modest landscape format of exactly 550 pixels wide and 300 pixels high (and please make sure that it remains clear enough even in these modest dimensions).

- Finally, during routine pre-acceptance checks, our data editors have raised the following queries regarding figures, data, and legends; I would appreciate if you briefly answered to them in the cover letter of your final submission, and made the requested text modifications with changes/additions highlighted via the "Track changes" option, to facilitate our final checking.
1. Please provide the exact p values in the legends of figures 6C, E, F
2. Please provide information related to 'n' in the legends of figures 6C, E, F; EV5 B, D
3. Please define the error bars in the legends of figures 6E, F; EV5 D.
4. Please add a scale bar and its definition for figures 6B, D; EV5A, C, E.

I am returning the manuscript to you for a final round of minor revision, solely to allow you to make these modifications and upload the revised files. Once we will have received them, we should hopefully be ready to accept the manuscript for publication.

With kind regards,

Hartmut

Hartmut Vodermaier, PhD
Senior Editor, The EMBO Journal
h.vodermaier@embojournal.org

*** PLEASE NOTE: All revised manuscript are subject to initial checks for completeness and adherence to our formatting guidelines. Revisions may be returned to the authors and delayed in their editorial re-evaluation if they fail to comply to the following requirements. As a first step please read our guidelines for revised submissions:
https://link.springer.com/journal/44318/submission-guidelines#cms-Revised-submissions

1) Every manuscript requires a Data Availability section (even if only stating that no deposited datasets are included). Primary datasets or computer code produced in the current study have to be deposited in appropriate public repositories prior to resubmission, and reviewer access details provided in case that public access is not yet allowed.

2) Each figure legend must specify
- size of the scale bars that are mandatory for all micrograph panels
- the statistical test used to generate error bars and P-values
- the type error bars (e.g., S.E.M., S.D.)
- the number (n) and nature (biological or technical replicate) of independent experiments underlying each data point
- Figures may not include error bars for experiments with n<3; scatter plots showing individual data points should be used instead.

3) Revised manuscript text (including main tables, and figure legends for main and EV figures) has to be submitted as editable text file (e.g., .docx format). We encourage highlighting of changes (e.g., via text color) for the referees' reference.

4) Each main and each Expanded View (EV) figure should be uploaded as individual production-quality files (preferably in .eps, .tif, .jpg formats). For suggestions on figure preparation/layout, please refer to our Figure Preparation Guidelines:
https://media.springernature.com/original/springer-cms/rest/v1/content/27825798/data/v1

5) Point-by-point response letters should include the original referee comments in full together with your detailed responses to them (and to specific editor requests if applicable), and also be uploaded as editable (e.g., .docx) text files.

6) Please complete our Author Checklist, and make sure that information entered into the checklist is also reflected in the manuscript; the checklist will be available to readers as part of the Review Process File.

7) All authors listed as (co-)corresponding need to deposit, in their respective author profiles in our submission system, a unique ORCiD identifier linked to their name. Please see our Guide to Authors for detailed instructions.

8) Please note that supplementary information at EMBO Press has been superseded by the 'Expanded View' for inclusion of additional figures, tables, movies or datasets; with up to five EV Figures being typeset and directly accessible in the HTML version of the article.

9) To facilitate reproducibilty and cross-laboratory adoption of methodologies, please structure the Materials & Methods section as outlined in our guide to authors, including a completed Reagents and Tools Table.

10) Digital image enhancement is acceptable practice, as long as it accurately represents the original data and conforms to community standards. If a figure has been subjected to significant electronic manipulation, this must be clearly noted in the figure legend and/or the 'Materials and Methods' section. The editors reserve the right to request original versions of figures and the original images that were used to assemble the figure. Finally, we generally encourage uploading of numerical as well as gel/blot image source data.

At EMBO Press we ask authors to provide source data for the main manuscript figures. You will receive a separate email with instructions for providing source data with your revised manuscript, including how to upload and organize the files.

In the interest of ensuring the conceptual advance provided by the work, we recommend submitting a revision within 3 months (23rd Nov 2026). Please discuss the revision progress ahead of this time with the editor if you require more time to complete the revisions. Use the link below to submit your revision:

Link Not Available

Referee #1:

The authors have addressed all my concerns. By the way, the labels, including "no effectors" and "no addition", in Figure 1G, 1H, 2B and 2F, should be checked again.

Referee #2:

The revision has largely addressed my comments and concerns.

Referee #3:

The authors have adequately addressed all my concerns and added a few figures to address this specific concern, and these new data strengthen the manuscript. The authors also addressed the questions from other reviewers, and I am happy to recommend the publication of this article in EMBO J.

EMBO Press Author Checklist

Corresponding Author Name: Jonathan N. Pruneda	
Journal Submitted to: The EMBO Journal	
Manuscript Number: EMBOJ-2024-119076	

USEFUL LINKS FOR COMPLETING THIS FORM
The EMBO Journal - Author Guidelines
EMBO Reports - Author Guidelines
Molecular Systems Biology - Author Guidelines
EMBO Molecular Medicine - Author Guidelines

Reporting Checklist for Life Science Articles (updated January
This checklist is adapted from Materials Design Analysis Reporting (MDAR) Checklist for Authors. MDAR establishes a minimum set of requirements in transparent reporting in the life sciences (see Statement of Task: 10.31222/osf.io/9sm4x). Please follow the journal's guidelines in preparing your
Please note that a copy of this checklist will be published alongside your article.

Abridged guidelines for figures
1. Data
The data shown in figures should satisfy the following conditions:
- the data were obtained and processed according to the field's best practice and are presented to reflect the results of the experiments in an accurate and unbiased manner.
- ideally, figure panels should include only measurements that are directly comparable to each other and obtained with the same assay.
- plots include clearly labeled error bars for independent experiments and sample sizes. Unless justified, error bars should not be shown for technical
- if n<5, the individual data points from each experiment should be plotted. Any statistical test employed should be justified.
- Source Data should be included to report the data underlying figures according to the guidelines set out in the authorship guidelines on Data

2. Captions
Each figure caption should contain the following information, for each panel where they are relevant:
- a specification of the experimental system investigated (eg cell line, species name).
- the assay(s) and method(s) used to carry out the reported observations and measurements.
- an explicit mention of the biological and chemical entity(ies) that are being measured.
- an explicit mention of the biological and chemical entity(ies) that are altered/varied/perturbed in a controlled manner.
- the exact sample size (n) for each experimental group/condition, given as a number, not a range;
- a description of the sample collection allowing the reader to understand whether the samples represent technical or biological replicates (including how many animals, litters, cultures, etc.).
- a statement of how many times the experiment shown was independently replicated in the laboratory.
- definitions of statistical methods and measures:
  - common tests, such as t-test (please specify whether paired vs. unpaired), simple χ2 tests, Wilcoxon and Mann-Whitney tests, can be unambiguously identified by name only, but more complex techniques should be described in the methods section;
  - are tests one-sided or two-sided?
  - are there adjustments for multiple comparisons?
  - exact statistical test results, e.g., P values = x but not P values < x;
  - definition of 'center values' as median or average;
  - definition of error bars as s.d. or s.e.m.

Please complete ALL of the questions below.
Select "Not Applicable" only when the requested information is not relevant for your study.

Materials

Newly Created Materials	Information included in the manuscript?	In which section is the information available? (Reagents and Tools Table, Materials and Methods, Figures, Data Availability Section)
New materials and reagents need to be available; do any restrictions apply?	Yes	Data Availability Section

Antibodies	Information included in the manuscript?	In which section is the information available? (Reagents and Tools Table, Materials and Methods, Figures, Data Availability Section)
For **antibodies** provide the following information:<br>- Commercial antibodies: RRID (if possible) or supplier name, catalogue number and or/clone number<br>- Non-commercial: RRID or citation	Yes	Reagents and Tools Table

DNA and RNA sequences	Information included in the manuscript?	In which section is the information available? (Reagents and Tools Table, Materials and Methods, Figures, Data Availability Section)
**Short novel DNA or RNA including primers, probes**: provide the sequences.	Yes	Reagents and Tools Table

Cell materials	Information included in the manuscript?	In which section is the information available? (Reagents and Tools Table, Materials and Methods, Figures, Data Availability Section)
**Cell lines:** Provide species information, strain. Provide accession number in repository **OR** supplier name, catalog number, clone number, and/**OR** RRID.	Not Applicable	
**Primary cultures:** Provide species, strain, sex of origin, genetic modification status.	Not Applicable	
Report if the cell lines were recently **authenticated** (e.g., by STR profiling) and tested for mycoplasma contamination.	Not Applicable	

Experimental animals	Information included in the manuscript?	In which section is the information available? (Reagents and Tools Table, Materials and Methods, Figures, Data Availability Section)
**Laboratory animals or Model organisms:** Provide species, strain, sex, age, genetic modification status. Provide accession number in repository **OR** supplier name, catalog number, clone number, **OR** RRID.	Yes	Reagents and Tools Table
**Animal observed in or captured from the field:** Provide species, sex, and age where possible.	Not Applicable	
Please detail **housing and husbandry conditions**.	Yes	Materials and Methods

Plants and microbes	Information included in the manuscript?	In which section is the information available? (Reagents and Tools Table, Materials and Methods, Figures, Data Availability Section)
**Plants:** provide species and strain, ecotype and cultivar where relevant, unique accession number if available, and source (including location for collected wild specimens).	Not Applicable	
**Microbes:** provide species and strain, unique accession number if available, and source.	Yes	Reagents and Tools Table

Human research participants	Information included in the manuscript?	In which section is the information available? (Reagents and Tools Table, Materials and Methods, Figures, Data Availability Section)
If collected and within the bounds of privacy constraints report on age, sex and gender or ethnicity for all study participants.	Not Applicable	

Core facilities	Information included in the manuscript?	In which section is the information available? (Reagents and Tools Table, Materials and Methods, Figures, Data Availability Section)
If your work benefited from core facilities, was their service mentioned in the acknowledgments section?	Not Applicable	

Design

Study protocol	Information included in the manuscript?	In which section is the information available? (Reagents and Tools Table, Materials and Methods, Figures, Data Availability Section)
If study protocol has been **pre-registered, provide DOI in the manuscript**. For clinical trials, provide the trial registration number **OR** cite DOI.	Not Applicable	
Report the **clinical trial registration number** (at ClinicalTrials.gov or equivalent), where applicable.	Not Applicable	

Laboratory protocol	Information included in the manuscript?	In which section is the information available? (Reagents and Tools Table, Materials and Methods, Figures, Data Availability Section)
Provide DOI OR other citation details if **external detailed step-by-step protocols** are available.	Not Applicable	

Experimental study design and statistics	Information included in the manuscript?	In which section is the information available? (Reagents and Tools Table, Materials and Methods, Figures, Data Availability Section)
Include a statement about **sample size** estimate even if no statistical methods were used.	Yes	Materials and Methods
Were any steps taken to minimize the effects of subjective bias when allocating animals/samples to treatment (e.g. **randomization procedure**)? If yes, have they been described?	Not Applicable	
Include a statement about **blinding** even if no blinding was done.	Not Applicable	
Describe **inclusion/exclusion criteria** if samples or animals were excluded from the analysis. Were the criteria pre-established?<br><br>If sample or data points were omitted from analysis, report if this was due to attrition or intentional exclusion and provide justification.	Not Applicable	
For every figure, are **statistical tests** justified as appropriate? Do the data meet the assumptions of the tests (e.g., normal distribution)? Describe any methods used to assess it. Is there an estimate of variation within each group of data? **Is** the variance similar between the groups that are being statistically compared?	Yes	Materials and Methods

Sample definition and in-laboratory replication	Information included in the manuscript?	In which section is the information available? (Reagents and Tools Table, Materials and Methods, Figures, Data Availability Section)
In the figure legends: state number of times the experiment was **replicated** in laboratory.	Yes	Figure Legends
In the figure legends: define whether data describe **technical or biological replicates**.	Yes	Figure Legends

Ethics

Ethics	Information included in the manuscript?	In which section is the information available? (Reagents and Tools Table, Materials and Methods, Figures, Data Availability Section)
Studies involving **human participants**: State details of **authority granting ethics approval** (IRB or equivalent committee(s), provide reference number for approval.	Not Applicable	
Studies involving **human participants**: Include a statement confirming that **informed consent** was obtained from all subjects and that the experiments conformed to the principles set out in the WMA Declaration of Helsinki and the Department of Health and Human Services Belmont Report.	Not Applicable	
Studies involving **human participants:** For publication of **patient photos**, include a statement confirming that consent to publish was obtained.	Not Applicable	
Studies involving experimental **animals**: State details of **authority granting ethics approval** (IRB or equivalent committee(s), provide reference number for approval. Include a statement of compliance with ethical regulations.	Not Applicable	
Studies involving **specimen and field samples:** State if relevant **permits** obtained, provide details of authority approving study; if none were required, explain why.	Not Applicable	

Dual Use Research of Concern (DURC)	Information included in the manuscript?	In which section is the information available? (Reagents and Tools Table, Materials and Methods, Figures, Data Availability Section)
Could your study fall under dual use research restrictions? Please check biosecurity documents and list of **select agents and toxins** (CDC): https://www.selectagents.gov/sat/list.htm	Not Applicable	
If you used a select agent, is the security level of the lab appropriate and reported in the manuscript?	Not Applicable	
If a study is subject to dual use research of concern regulations, is the name of the **authority granting approval and reference number** for the regulatory approval provided in the manuscript?	Not Applicable	

Reporting

The MDAR framework recommends adoption of discipline-specific guidelines, established and endorsed through community initiatives. Journals have their own policy about requiring specific guidelines and recommendations to complement MDAR.

Adherence to community standards	Information included in the manuscript?	In which section is the information available? (Reagents and Tools Table, Materials and Methods, Figures, Data Availability Section)
State if relevant guidelines or checklists (e.g., **ICMJE, MIBBI, ARRIVE, PRISMA**) have been followed or provided.	Not Applicable	
For **tumor marker prognostic studies,** we recommend that you follow the **REMARK** reporting guidelines (see link list at top right). See author guidelines, under 'Reporting Guidelines'. Please confirm you have followed these guidelines.	Not Applicable	
For **phase II and III randomized controlled trials**, please refer to the **CONSORT** flow diagram (see link list at top right) and submit the CONSORT checklist (see link list at top right) with your submission. See author guidelines, under 'Reporting Guidelines'. Please confirm you have submitted this list.	Not Applicable	

Data Availability

Data availability	Information included in the manuscript?	In which section is the information available? (Reagents and Tools Table, Materials and Methods, Figures, Data Availability Section)
Have **primary datasets** been deposited according to the journal's guidelines (see 'Data Deposition' section) and the respective accession numbers provided in the Data Availability Section?	Yes	Source data provided
Were **human clinical and genomic datasets** deposited in a public access-controlled repository in accordance to ethical obligations to the patients and to the applicable consent agreement?	Not Applicable	
Are **computational models** that are central and integral to a study available without restrictions in a machine-readable form? Were the relevant accession numbers or links provided?	Not Applicable	
If publicly available data were reused, provide the respective **data citations in the reference list**.	Not Applicable	
