# Supplementary material for: A functional screen for bacterial ubiquitin regulation identifies an unusual Pseudomonas aeruginosa E3 ligase
Source: EMBO J. 2026 Sep 28;45(21):7489–514. doi: 10.1038/s44318-026-00917-7 (PMC13728172; doi:10.1038/s44318-026-00917-7)
Supplement: Supplementary file 2 [file 44318_2026_917_MOESM8_ESM.pdf]

Expanded View Figures

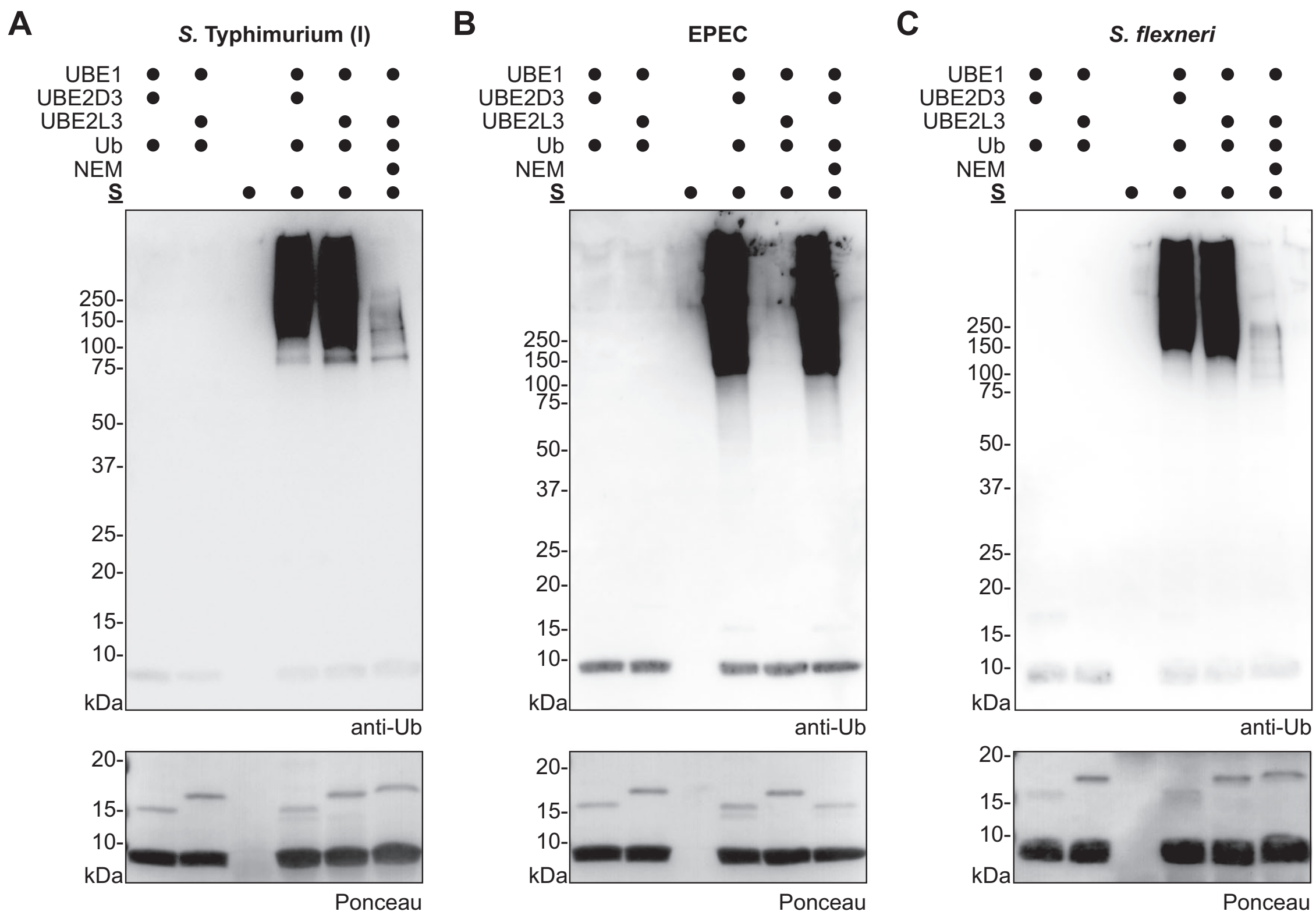

Figure EV1. A functional screen for ubiquitin regulation.

(**A**) E3 ligase assays combining the indicated reaction components with the SPI-I secreted fraction from *S.* Typhimurium, with and without prior treatment with NEM. Reactions were resolved by SDS-PAGE and visualized by anti-Ub western blot. (**B**) As in (**A**), for the secreted fraction from EPEC. (**C**) As in (**A**), for the secreted fraction from *S. flexneri*.

© The Author(s)

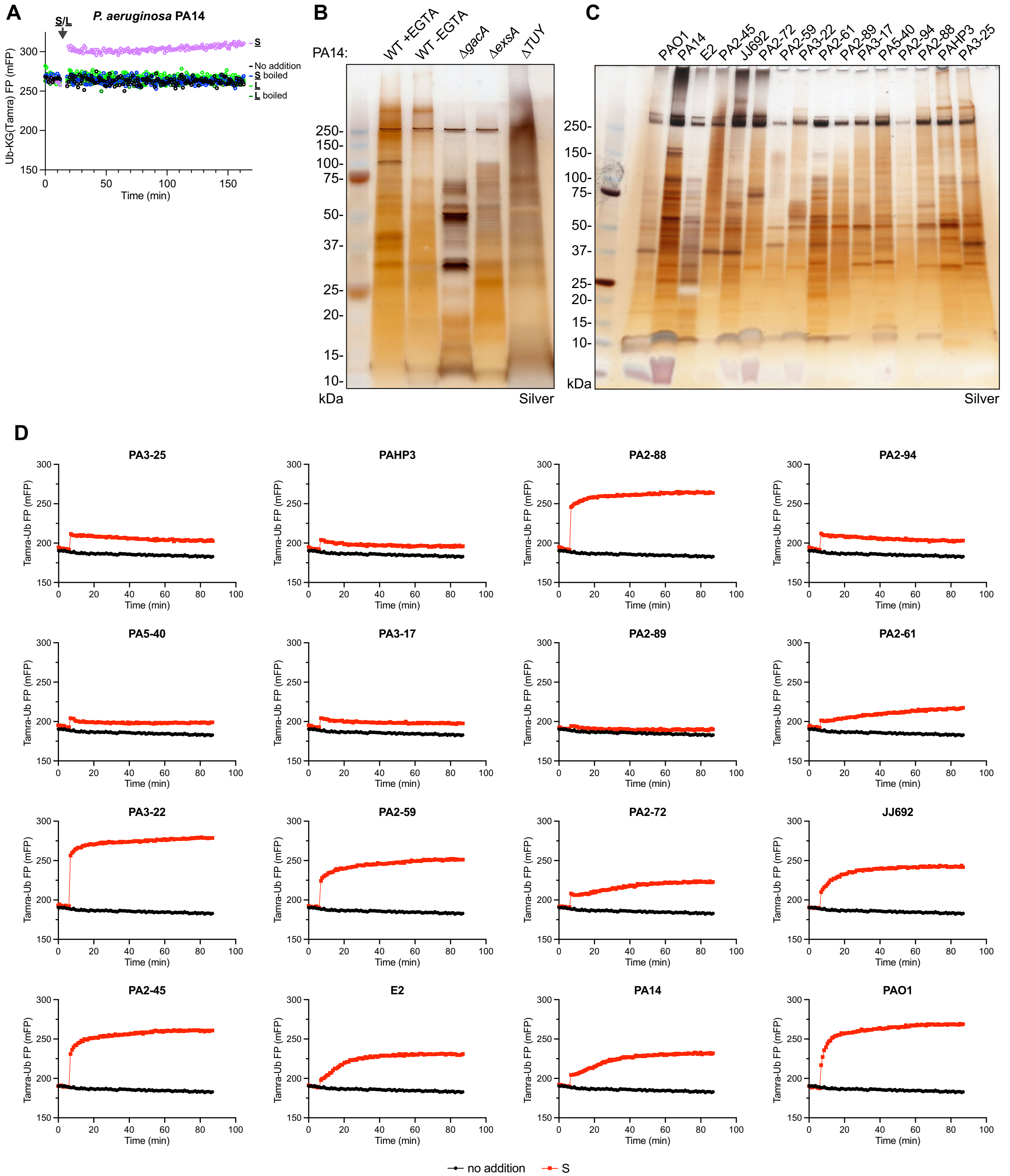

Figure EV2. Detection of E3 ligase activity secreted by *P. aeruginosa*.

(**A**) Representative FP traces monitoring the Ub-KG(Tamra) DUB substrate following addition of the indicated pools of *P. aeruginosa* protein. (**B**) Silver-stained SDS-PAGE analysis of secreted fractions generated from the indicated PA14 mutant strains, or in the absence of EGTA stimulation. (**C**) Silver-stained SDS-PAGE analysis of secreted fractions generated from the indicated *P. aeruginosa* clinical isolates. (**D**) Representative FP traces monitoring the Tamra-Ub ligase substrate following the addition of secreted fractions from the indicated *P. aeruginosa* clinical isolates.

 © The Author(s)

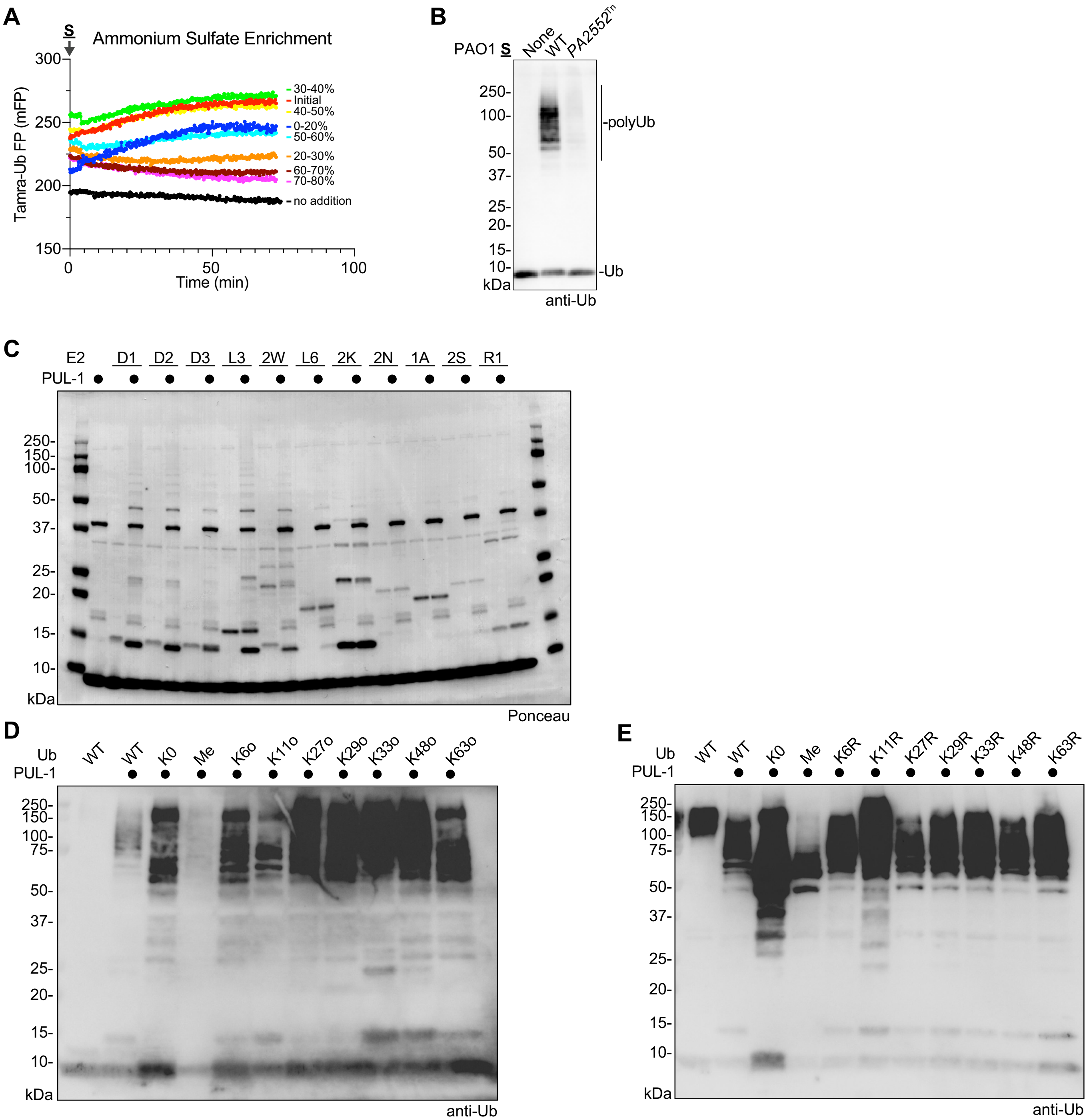

F

Protein	Site
PUL-1	K105
PUL-1	K136
PUL-1	K271
Ub	K6
Ub	K11
Ub	K27
Ub	K33
Ub	K48
Ub	K63

© The Author(s)

Figure EV3. Identification of a *P. aeruginosa* E3 ligase.

(**A**) Representative FP traces monitoring the Tamra-Ub ligase substrate following the addition of the indicated ammonium sulfate fractions of PA14 secreted protein. (**B**) E3 ligase assays for secreted fractions generated from PAO1 wild-type or the *PA2552*^{Tn} mutant strain. Reactions were resolved by SDS-PAGE and visualized by anti-Ub western blot. (**C**) E3 ligase assays for recombinant PUL-1 and the indicated panel of E2 enzymes. Ponceau-stained visualization of Fig. 4D. (**D**) E3 ligase assays for recombinant PUL-1 and Lys-less (K0), methylated, or the indicated panel of K-only Ub mutants. Reactions were resolved by SDS-PAGE and visualized by anti-Ub western blot. (**E**) E3 ligase assays for recombinant PUL-1 and Lys-less (K0), methylated, or the indicated panel of K-to-R Ub mutants. Reactions were resolved by SDS-PAGE and visualized by anti-Ub western blot. (**F**) Ubiquitination sites identified by mass spectrometry following an in vitro PUL-1 ligase reaction.

© The Author(s)

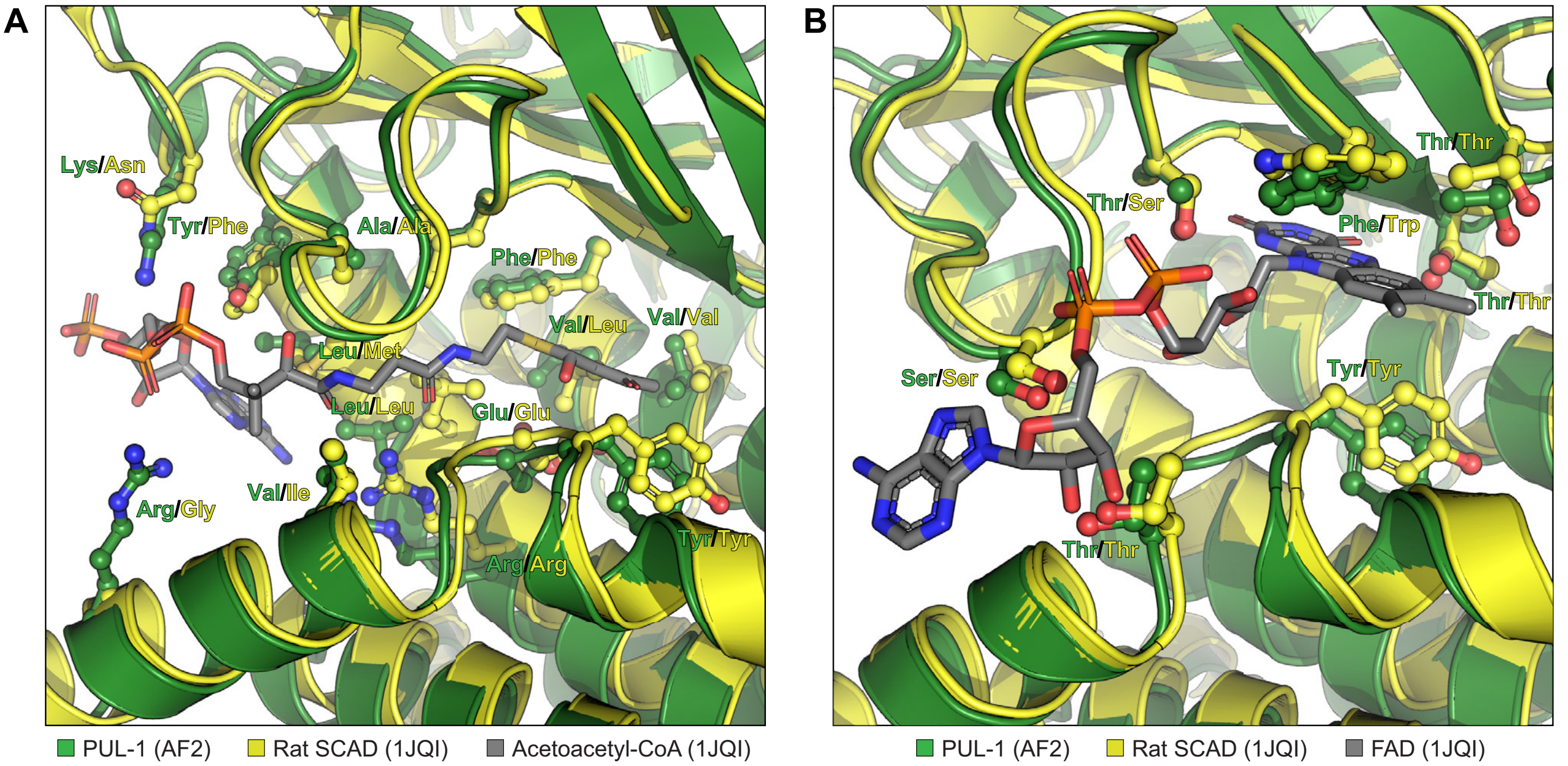

C

Strain	PUL-1 sequence substitutions (relative to PAO1)
PAO1	-
PA14	A372V
PA2-45	None
PA2-59	None
PA2-61	E7D, T220A
PA2-72	None
PA2-88	None
PA2-89	None
PA2-94	None
PA3-17	None
PA3-22	None
PA3-25	None
PA5-40	None
PAHP3	None
E2	R260C
JJ692	None

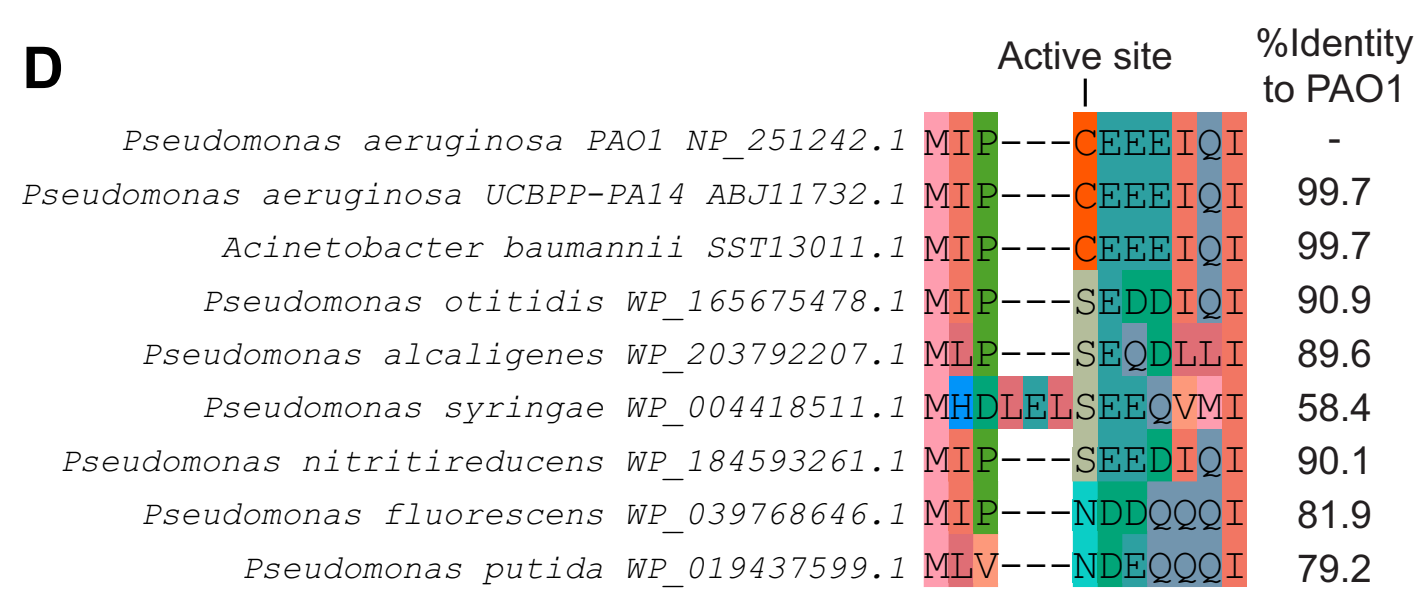

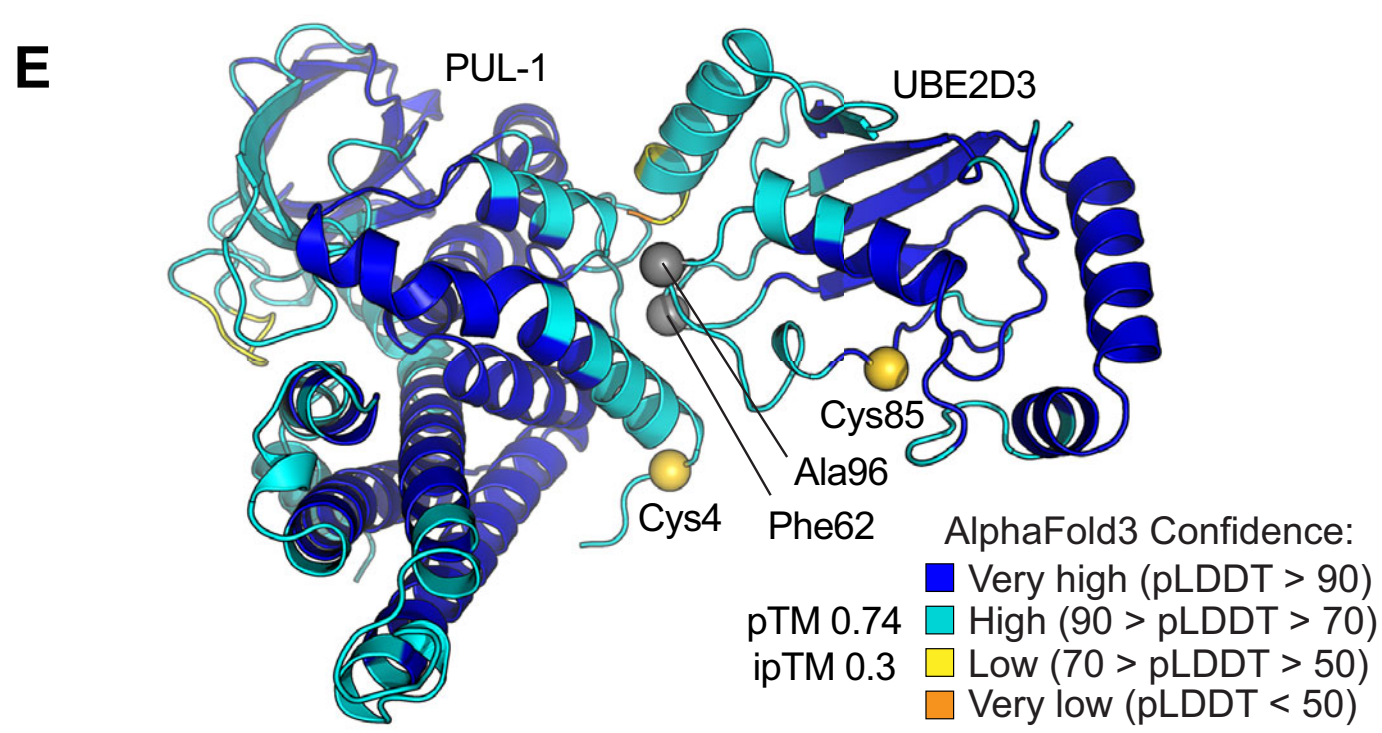

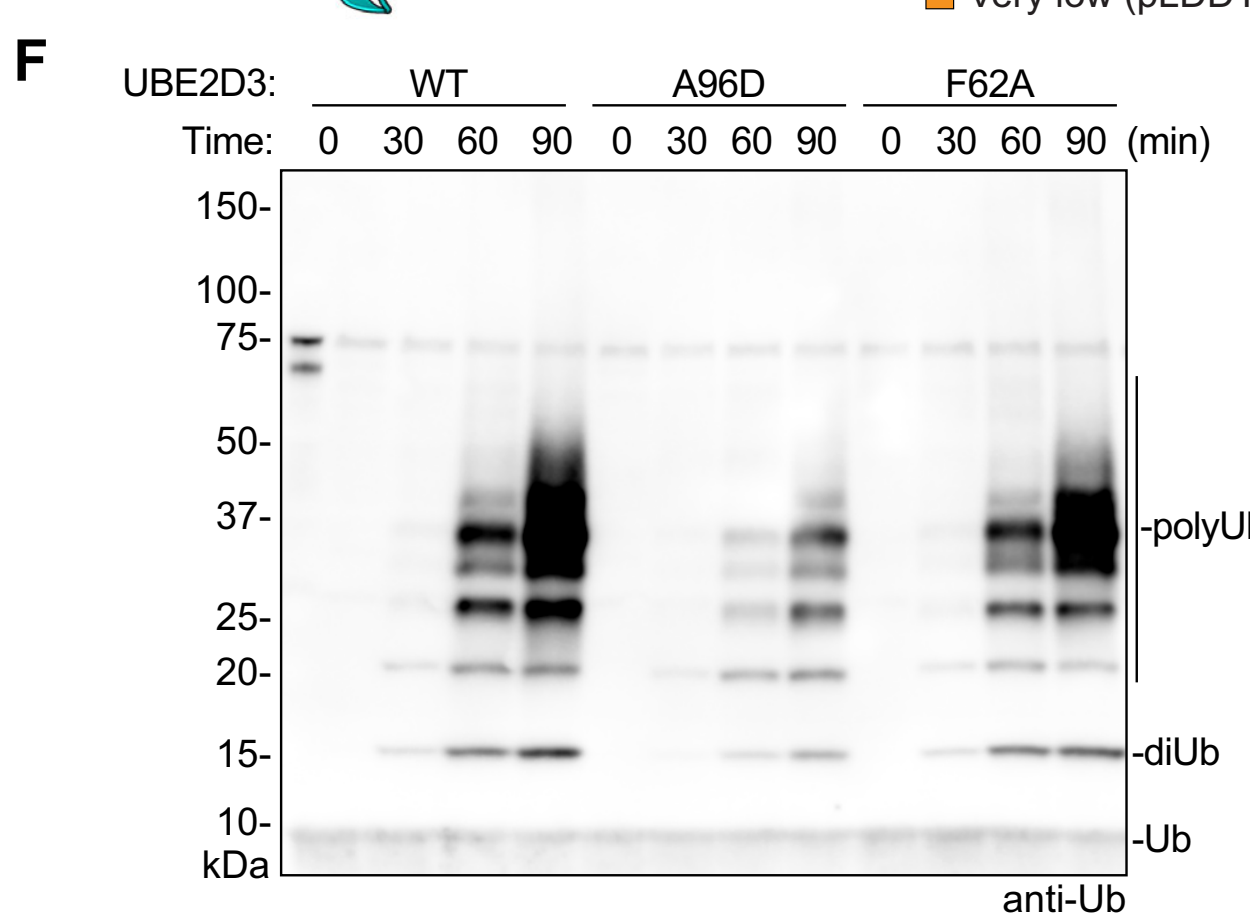

Figure EV4. Structural analysis of the PUL-1 ligase fold.

(**A**) Structural overlay of the mitochondrial short-chain specific acyl-CoA dehydrogenase (SCAD) from rat (yellow, PDB: 1JQI), with acetoacetyl-CoA bound (gray sticks), and the PUL-1 AlphaFold2 model (green). Residues within the acyl-CoA-binding pocket are shown in ball-and-stick for both enzymes. (**B**) Structural overlay of the mitochondrial short-chain specific acyl-CoA dehydrogenase (SCAD) from rat (yellow, PDB: 1JQI), with FAD bound (gray sticks), and the PUL-1 AlphaFold2 model (green). Residues within the FAD-binding pocket are shown in ball-and-stick for both enzymes. (**C**) Conservation of PUL-1 orthologues among all *P. aeruginosa* clinical isolates is presented in Fig. 2F. Amino acid substitutions relative to PAO1 are listed. All remaining amino acids, including the active site, are perfectly conserved. (**D**) Sequence alignment of PUL-1 orthologues, focused on the region surrounding Cys4 of PAO1. (**E**) AlphaFold3 model of the PUL-1:UBE2D3 complex, color by pLDDT confidence scores. Catalytic Cys residues of PUL-1 and UBE2D3 are indicated in gold spheres, and residues at the E3-interacting surface of UBE2D3 selected for mutational analysis are indicated in gray spheres. (**F**) E3 ligase assay for PUL-1 with wild-type or the indicated UBE2D3 mutants. Reactions were resolved by SDS-PAGE and visualized by anti-Ub western blot.

© The Author(s)

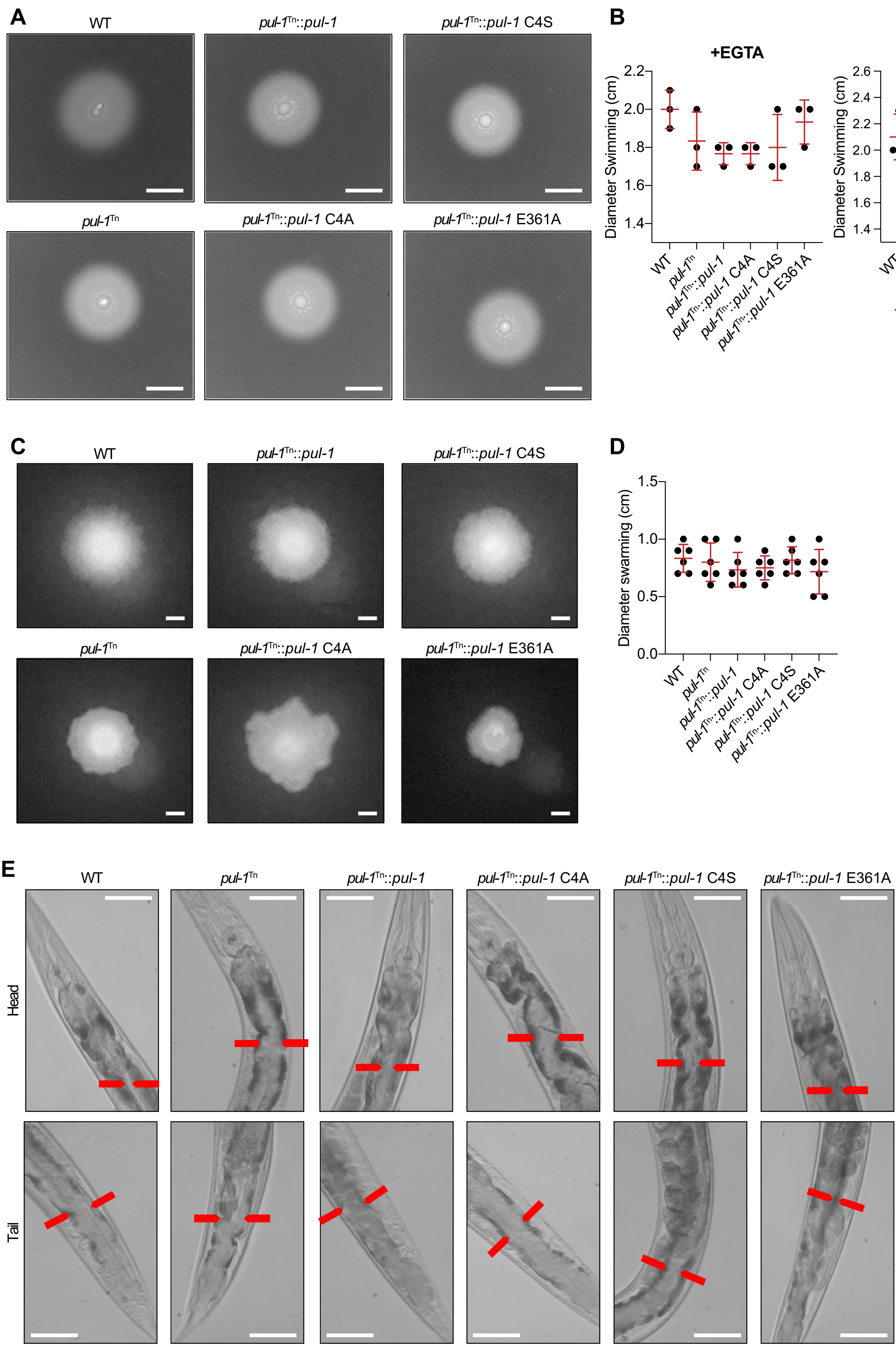
A
WT
pul-1Tn::pul-1
pul-1Tn::pul-1 C4S
pul-1Tn
pul-1Tn::pul-1 C4A
pul-1Tn::pul-1 E361A
B
+EGTA
-EGTA
Diameter Swimming (cm)
WT
pul-1Tn
pul-1Tn::pul-1
pul-1Tn::pul-1 C4A
pul-1Tn::pul-1 C4S
pul-1Tn::pul-1 E361A
C
D
Diameter swarming (cm)
E
Head
Tail

Figure EV5. Secreted PUL-1 ligase activity modulates *P. aeruginosa* virulence.

(**A**) Representative images of *P. aeruginosa* swimming for WT PAO1 and the indicated *pul-1*^{Tn} mutant strains. Scale bars correspond to 1 cm. (**B**) Quantification of (**A**), under conditions with and without EGTA. Mean values and standard deviation are indicated in red for $n = 3$ biological replicates. (**C**) Representative images of *P. aeruginosa* swarming for WT PAO1 and the indicated *pul-1*^{Tn} mutant strains. Scale bars correspond to 2 mm. (**D**) Quantification of (**C**). Mean values and standard deviation are indicated in red for $n = 6$ biological replicates. (**E**) Representative images of *C. elegans* intestinal bloating near the head and tail, following infection with WT PAO1 or the indicated *pul-1*^{Tn} mutant strains. The intestinal lumen diameter is indicated by red arrows. Scale bars correspond to 200 μm. Images presented in Fig. 6D are provided again for comparison.

 © The Author(s)
