# Supplementary material for: A functional screen for bacterial ubiquitin regulation identifies an unusual Pseudomonas aeruginosa E3 ligase
Source: EMBO J. 2026 Sep 28;45(21):7489–514. doi: 10.1038/s44318-026-00917-7 (PMC13728172; doi:10.1038/s44318-026-00917-7)

Extended Fig 5. Structural analysis of the PUL-1 ligase fold

5H **5I**

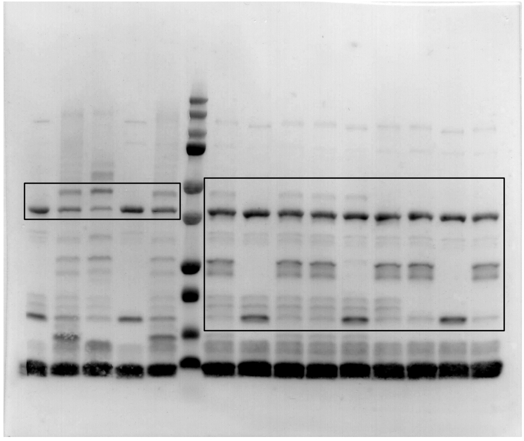

Supplement: Supplementary file 7 [file 44318_2026_917_MOESM6_ESM.zip › Figure 5/5H-I/5H-I.pdf]
